# School Social Workers’ Reports of Differences in Policies and Practices in Trauma-Informed and Non-Trauma-Informed Schools

**DOI:** 10.3390/bs14110991

**Published:** 2024-10-24

**Authors:** Kate R. Watson, Ron Avi Astor, Gordon P. Capp, Rami Benbenishty

**Affiliations:** 1 Luskin School of Public Affairs, University of California, Los Angeles, CA 90095, USA; rastoravi@ucla.edu; 2 School of Education and Information Studies, University of California, Los Angeles, CA 90095, USA; 3 Department of Social Work, California State University, Fullerton, CA 92831, USA; gcapp@fullerton.edu; 4 The Paul Baerwald School of Social Work and Social Welfare, The Hebrew University of Jerusalem, Jerusalem 9190501, Israel; ramibenben@gmail.com; 5 Education and Social Sciences School, Universidad Andrés Bello, Santiago 8370146, Chile

**Keywords:** trauma informed, school climate, school social work, school safety

## Abstract

This study explored trauma-informed schools from the perspective of social workers, documenting the reported practices and policies associated with trauma-informed approaches in U.S. schools. Survey data from 538 school social workers were analyzed to investigate the differences in policies and practices between schools identified as trauma informed and not. Logistic regression analyses examined whether the presence of specific school practices and policies was associated with the identification of a school as trauma informed. Of a wide array of programs and policies that may be present in trauma-informed schools, only the presence of trauma training and resources for secondary traumatic stress were key predictors of social workers’ identification of a school as trauma informed. The implementation of trauma training has long been the primary focus of trauma-informed approaches in schools. Should commitment to trauma-informed approaches endure, we recommend moving beyond training and secondary traumatic stress resources to deepen the field’s focus on implementing trauma-informed practices and policies at all organizational levels. We also recommend that future research looks carefully at how some school safety and trauma-informed approaches may be incompatible and the extent to which trauma-informed approaches improve or detract from children’s educational experiences and outcomes.

## 1. Introduction

The U.S. federal government provides free, universal, public K–12 education. However, policies that affect the day-to-day management of schools, their priorities, and curricula are set at the local or state level [1]. As a result, schools in different districts, cities, or states can look very different in terms of their teaching and disciplinary practices, available programs, and schoolwide policies [2]. As one example, only 27 U.S. states explicitly prohibit the use of corporal punishment—state-sanctioned violence that is documented by global research as harmful and traumatizing for students [3,4]—as school discipline [5].

K–12 education funding is also allocated by localities and states [1]. Often, these funds are earmarked for specific purposes or are time limited, which affects schools’ ability to create holistic, long-term strategies to serve the educational and socioemotional needs of children and communities. As a result, schools tend to adopt short-term strategies or purchase programs in response to their priorities, e.g., implementing a social and emotional learning (SEL) curriculum to enhance students’ interpersonal skills or ALICE training (which stands for alert, lockdown, inform, counter, and evacuate) to promote staff and student safety during an active shooter event. Such choices create a fractured school system with duplication of services, inefficient spending, and confusion among staff, students, and families about what resources are available and how to access them [6,7]. Many academic researchers have promoted remedies to these problems by suggesting a focus on whole-school approaches [6,7,8], positive school climate [9,10], and school missions that include issues of care, welcoming, and response to trauma [11].

For the past decade—due to growing recognition of the prevalence of trauma in our society and its impact on children’s development and learning [12]—schools have increasingly prioritized the implementation of trauma-informed approaches [13]. Many organizations have proposed trauma-informed conceptual frameworks [14,15,16,17,18]. To date, the few studies that have explored trauma-informed school interventions have focused mainly on trauma training for staff and access to trauma-specific clinical treatments like trauma-focused cognitive behavioral therapy [19]. It is unclear what most schools are doing in practice to create trauma-informed environments. Studies that explore practice and policy differences between schools identified as trauma informed and not trauma informed are limited, if not nonexistent.

School social workers are ideal respondents to questions about trauma-informed approaches in schools because across the United States, they often provide frontline mental health services to students and their families. School social workers are typically responsible for organizing SEL in schools and may provide individual case planning for children with emotional and behavioral health issues. They also are one of few school professionals with advanced training in trauma [20,21].

This study was based in part on the first author’s dissertation [22] and assessed how school policies and practices differed between schools identified as trauma informed and not trauma informed. Based on the extant research literature, it is unclear if there are major differences between trauma-informed and non-trauma-informed schools. It is also possible that due to large influxes of federal and local funding resulting from school shootings, a wide array of law enforcement, hardening, zero tolerance, and supportive practices coexist in the same school. Given the lack of a standardized measure or designation to indicate whether a school is trauma informed, this study sought to understand the relationship between current school policies and practices and a school’s identification as trauma informed. The research questions for this article were: Do school social workers report differences in the policies and practices between trauma-informed and non-trauma-informed schools? Can the presence of certain policies or practices indicate whether a school is trauma informed?

## 2. Literature Review

### 2.1. The Role of Social Workers in Schools

The views of social workers are critical to any discussion about trauma-informed approaches in schools. In many states, school social workers serve as frontline mental health providers, and throughout the United States, their caseloads include children with a wide range of behavioral health issues, including trauma responses [20,21]. As mental health professionals, social workers are among the few school staff members trained in trauma, its effects, and prevention and intervention, regardless of whether the school they work in is trauma informed. Thus, school social workers are perhaps the best suited staff members to determine and report on whether a school is trauma informed or not. In this study, we sought to understand the perceptions of school social workers through reported differences in policies and practices in trauma-informed and non-trauma-informed schools.

### 2.2. Brief Summary of the Evolution of Trauma-Informed Approaches in Schools

During the past two decades, recognition of the prevalence of trauma in society and how it can affect children’s learning and development has increased [12]. Federal legislation, such as Every Student Succeeds Act [23], SUPPORT for Patients and Communities Act [24], and Bipartisan Safer Communities Act [25], calls for school-based mental health services and staff training rooted in evidence-based, trauma-informed practice and has provided funding for such purposes. As a result, schools have increasingly prioritized the implementation of trauma-informed approaches despite limited evidence supporting them [13,19,26,27].

It also remains unclear whether schools’ trauma-informed interventions are integrated with the broader purpose of schools or if certain components considered trauma informed are potentially harmful [19]. Currently, many evidence-based programs have described themselves as trauma informed [27,28], but it is not clear if these approaches are aligned with the foundations of trauma-informed conceptual frameworks. In addition, there has been some discussion in the educational literature about the deficit-oriented and negative implications of labeling a school as trauma informed [29,30,31].

Since the concept of a trauma-informed organization was proposed by clinicians Maxine Harris and Roger Fallot [32], many other practitioners and agencies have suggested definitions and components for trauma-informed organizations and schools. One of the most prominent models was put forth by the Substance Abuse and Mental Health Services Administration [33], which defines a trauma-informed program, organization, or service as one that meets the following criteria:

Realizes the widespread impact of trauma and understands potential paths for recovery; recognizes the signs and symptoms of trauma in clients, families, staff, and others involved with the system; responds by fully integrating knowledge about trauma into policies, procedures, practices; and seeks to actively resist re-traumatization.

(p. 9)

Hanson and Lang [34] identified three primary domains for operationalizing trauma-informed care in organizations: (a) the implementation of trauma-informed trainings and workforce development, (b) the presence of trauma-specific services and treatments, and (c) adaptations to the organizational environment including an emphasis on safety, staff collaboration, and written policies related to trauma. Subsequently, researchers have used these three domains to assess the state of trauma-informed approaches in schools.

A 2019 Campbell Collaboration review determined that there was little to no empirical evidence for trauma-informed approaches in schools despite their popularity and that it was unclear whether the cost–benefit tradeoffs were justified [19]. Several other authors have explained that confusion remains about the essential components and best way to implement trauma-informed approaches in schools as well as what outcomes should be expected [26,27,35]. A more recent systematic review found only four studies that reported results for whole-school, trauma-informed approaches [26]. Apart from one study [36], interventions were employed at small schools with limited participants.

### 2.3. State-Level Policies Related to Trauma-Informed Schools in the United States

Conceptualizations for trauma-informed schools identify several common school policies and practices as relevant to the implementation of trauma-informed approaches. For example, the multitiered system of support (MTSS), SEL programs, school safety, and school climate initiatives have aligned with the concept of trauma-informed schools [14,16,17,18,37,38,39]. It is unclear if many of these pre-existing evidence-based programs fully align with trauma-informed conceptualizations. However, many schools and social workers will select these programs to address trauma-related issues in their school [21,40,41]. Therefore, in the pupil personnel literature (e.g., social work, psychology, counseling, and nursing), the following programs are among the most frequently discussed. A review of these approaches is important because at first glance, these programs may not be seen or understood as trauma informed without further explanation. Yet in recent years, many practitioners referred to these approaches interchangeably with trauma-informed interventions [21,40,42,43].

#### 2.3.1. MTSS

MTSS is a standard framework for delivering academic and behavioral interventions in U.S. schools. Rooted in a public health intervention framework, MTSS provides three tiers of support: (1) universal, (2) supplemental support as needed, and (3) intensive support where indicated. An umbrella term, MTSS includes academic programs like response to intervention and behavioral modification programs like positive behavioral intervention and support (PBIS) [44]. Some researchers have called for further integration of trauma-informed approaches and MTSS [19,45].

#### 2.3.2. School Climate Programs

Positive school climate has been linked to academic and behavioral benefits for students [46,47]. As such, school climate programs have become a common intervention to improve schools and enhance their safety [48]. Commonly identified dimensions of school climate include the academic environment, physical and institutional structure, safety, and relationship quality, including belonging [46,47]. Several aspects of school climate are reflected in trauma-informed approaches, including an emphasis on physical and psychological safety and positive relationships.

#### 2.3.3. SEL Programs

SEL programs help students gain awareness of and manage their emotions, understand interpersonal communication, build positive relationships, and make responsible decisions. As of 2022, 27 states had adopted SEL standards for K–12 education, and all states had adopted them for pre-K [37]. Although popular, SEL programs are not without criticism. Some parents believe schools should focus on teaching academic skills [49]. Also, many SEL programs ignore systemic factors like racism, historical and intergenerational trauma, and socioeconomic inequalities that affect young people’s socioemotional development, causing school staff to ignore important differences in children’s experiences and perpetuate inequities [50,51]. The COVID-19 pandemic heightened awareness of trauma in schools, leading some educators and school professionals to explore SEL programs as a potential solution. This exploration resulted in a blurring of the lines between SEL programs and school-based trauma response [21,52].

#### 2.3.4. Teaching Materials That Reflect a School’s Student Population

In the last decade, the racial and ethnic composition of U.S. public schools has shifted. Whereas White students represented the majority of public-school enrollment in 2010, by 2021, the percentage of White students had decreased to 45%. At the same time, the percentage of students from Hispanic, Asian, and multiracial backgrounds grew, and the percentage of students from Black and Native American families stayed about the same [53]. These shifting demographics call for increased attention and commitment toward cultural awareness and humility to ensure students from all backgrounds can succeed in the classroom [54,55]. To create an inclusive environment that promotes student well-being, belonging, and psychological safety—all important elements of trauma-informed approaches—it is essential to understand students’ and families’ contexts and cultural heritage [11,56,57,58].

#### 2.3.5. Security Measures to Promote Physical Safety

Starting in the 1980s, in response to increasing public concern about school safety and crime, U.S. schools dramatically expanded the presence of security measures, including surveillance systems, metal detectors, and school resource officers (i.e., police officers who serve schools) [59,60,61]. Decades of research have shown that security measures can threaten the development and sustainment of positive teacher–student relationships [62,63] and contribute to harsh discipline and disciplinary inequities, particularly for students of color [64,65,66,67,68].

In the trauma-informed literature, harsh discipline and exclusionary practices are antithetical to the trauma-informed approach [69,70]. Although law enforcement measures have not been included in the formal definitions or conceptualizations of trauma-informed schools, they do closely align with concepts in school safety. It is possible that some practitioners and researchers approach trauma-informed interventions in the larger framework of school safety interventions. As mentioned, there is some evidence that hardening, policing, and zero-tolerance interventions are counter-indicated with traditional trauma-informed approaches [69]. Yet, if trauma-informed programs are seen as additional tools in the larger school safety framework, it is possible that practitioners could report the existence of trauma-informed and hardening approaches in the same schools.

#### 2.3.6. Equitable Discipline in Schools

Equitable discipline practices have been growing in schools as a response to mid-1990s to 2000s punitive and exclusionary practices that created significant inequities between White students and students of color. Restorative justice is one such approach that is rooted in Indigenous beliefs and teaches students to repair harm after conflict instead of taking a punitive or shame-based approach [71]. Equitable discipline practices are in better alignment with trauma-informed approaches than harsh discipline and exclusionary practices [11,58,70].

#### 2.3.7. Trauma-Informed and Supportive School Policies

Several school policies are related to trauma awareness and clinical interventions to trauma. A school may provide trauma training for staff, trauma psychoeducation for students and their families, screening for trauma symptoms or adverse childhood experiences, trauma-specific treatments such as individual counseling or group therapy, and resources for secondary traumatic stress (STS) to support staff. These trauma-focused policies and practices are typically situated in a broader commitment to student mental health and well-being. Although not trauma specific, other policies that could be considered supportive of a trauma-informed approach include the presence of SEL, equitable school discipline, restorative and de-escalation practices, adapting course content to reflect student diversity, and removing potentially triggering materials from curricula [16].

#### 2.3.8. School Policies and Practices During the COVID-19 Pandemic

The COVID-19 pandemic led to several practice and policy changes as schools closed in person and began providing academic and behavioral health services online [40]. Among the practice and policy changes associated with this period were pandemic-specific changes, including mandatory masking and vaccinations, and changes to the availability of academic instruction such as online or hybrid class options and expanded teaching hours. Changes were enabled due to an influx of federal funding [72]. Although many policy and practice changes during the pandemic related to public health concerns, they also sought to support the basic needs and worsening mental health of children and families [73]. These latter policies related to the provision of trauma-informed approaches in schools.

## 3. Methods

### 3.1. Population and Study Samples

The sample (*N* = 538) for this study was recruited from a population of school social workers across the United States through professional organizations, including the National Association of Social Workers, School Social Work Association of America, School Social Work Network, and other state-level associations. The research team collaborated with the professional organizations, which distributed a link to an anonymous online survey developed by researchers from UCLA; California State University, Fullerton; and Hebrew University in Jerusalem. Some survey participants had responded to a prior survey deployed by the same research team in 2020 and were recruited to complete the current survey because they had provided their email addresses for follow up. The survey was administered between March and June 2022.

Participants were from 43 U.S. states and the District of Columbia, with the highest concentrations in Illinois (18.3%), California (16.8%), Michigan (5.8%), and Connecticut (5.4%). Table 1 provides detailed demographics for the sample. Most participants (90.7%) self-identified as female, 7.2% as male, 0.4% as gender nonconforming, and others chose not to disclose. The sample primarily identified as White or Caucasian (72.7%), followed by 11.7% as Hispanic or Latinx, 7.4% as Black or African American, 4.3% as multiracial, 0.7% as Asian American, 0.2% as Native Hawaiian or Pacific Islander, 0.2% as Native American or Alaska Native, and others chose not to disclose. Practicing school social workers were the primary respondents (89.2%); 1.5% identified as district social work supervisors, 0.9% were heads of social work services in a district, 0.6% were school-based social work contractors, and the remaining held other school-based positions or chose not to disclose. Participants’ years of social work experience ranged from less than 1 (4.3%) to more than 20 (23.7%), with a mean of more than 10 years. Participants served in various grade levels, including preschool (17.1%), elementary (57.6%), middle or junior high (48.3%), and high school (44.8%) or alternative schools (12.8%). Many participants reported serving in multiple schools simultaneously.

School characteristics reported by participants are presented in Table 2. Participants worked in suburban (43.3%), urban (38.1%), and rural (18.2%) districts across the United States. The Midwest region had the largest representation (35.6%), followed by the West (25.0%), Northeast (20.5%), and South (18.9%). Participants reported working in high-need schools. The estimated percentage of students who qualified for free or reduced-price lunch was 63.8% (*SD* = 28.4%). Participants estimated that more than half of their students were from historically marginalized populations (*M* = 55.7%, *SD* = 30.7%); almost 1 in 5 district students dropped out (*M* = 19.5%, *SD* = 18.1%); and only about half of students entered college (*M* = 55.5%, *SD* = 22.1%).

### 3.2. Instrument and Ethics

Survey questions were developed by the research team to understand the needs of school staff, students, and families during the 2021–2022 school year and the relationship between those needs and the extant models for trauma-informed care in schools. The instrument included both closed and open-ended questions about schools’ programs and policies, including those related to the ongoing COVID-19 pandemic, and solicited school social workers’ views about the climate of their school environment. The research team received approval from the institutional review board at the University of California, Los Angeles, and partner organizations completed their own internal ethics review processes.

### 3.3. Measures

#### 3.3.1. Personal Characteristics

Respondents were asked to report their professional role (i.e., school social worker, district supervisor, head of services in a district, school-based contractor, or other), state or U.S. territory in which they practice, community setting (i.e., urban, suburban, rural), types of schools they support (e.g., preschool, elementary, etc.), and number of years they practiced as a school social worker. They were also asked to report gender and race and ethnicity.

#### 3.3.2. School Characteristics

Participants were asked to report on the characteristics of their school district, including their estimates of the percentage of students eligible for free or reduced-price lunch, from historically marginalized populations, who drop out, and who enter college.

#### 3.3.3. Report of Whether School Is Trauma-Informed or Not

Participants were asked to choose one school with which they work and indicate whether that school was considered a trauma-informed setting. The selection of one school enabled reporting of individual policies and practices in that school.

#### 3.3.4. School Practices and Policies Present

Participants were asked to identify practices or policies in the school they chose during the 2021–22 academic year. They reviewed two lists of practices and policies offering 33 options, including “none of the above” and space for a brief written response to suggest additional policies. The first list featured typical school policies and practices, including SEL skills training, school climate programs, and a commitment to creating a safe, supportive learning environment for all students. Some options were related to increasing the physical safety of schools through metal detectors or school resource officers or police presence. Other options were specific to trauma, including screening for adverse childhood experiences or post-traumatic stress disorder (PTSD), trauma training for staff, or trauma psychoeducation for students or parents. Due to the time in which data were collected (spring 2022), a second list of policies and practices specific to COVID-19 such as mandatory vaccination and social distancing was also offered.

A single dichotomous variable, participant reports of whether their school is trauma informed or not, was the dependent variable. Participant reports of school policies present during the current school year, including those that were COVID-19 specific, were independent variables. Participant demographics and school characteristics served as control variables.

### 3.4. Analysis

To compare practices and policies present in schools that social workers identified as trauma informed with schools they indicated were not trauma informed, we first conducted several chi-square tests of independence. The analyses were conducted on two groups differentiated by the dependent variable (i.e., schools that social workers identified as trauma informed vs. those that were not). Through our analyses, we sought to identify differences between trauma-informed and non-trauma-informed schools associated with policies and practices and not with the socioeconomic or demographic characteristics of the schools or social workers. Due to the small sample sizes of some racial and ethnic and gender groups (i.e., Asian, Native Hawaiian, Native American, and trans or nonbinary individuals), comparisons were only possible between Black, Hispanic, White, and other groups for race and ethnicity, and between male and female for gender. Dummy codes were created for race and ethnicity, with other serving as the reference group. Male served as the reference group for gender.

Exploratory factor analyses of policies and practices using Pearson and polychoric correlations were conducted to determine if individual policies and practices could be factored to simplify regression models. No factor structure incorporated all policies and significantly reduced the variables; therefore, all policies and practices were entered independently into regression analyses.

To determine if the presence of certain policies and practices could predict whether a school would be identified as trauma informed, logistic regressions were conducted. Due to the large number of independent variables, we conducted: (a) one regression to compare general school policies against school type (trauma informed or not); and (b) a second regression to compare COVID-19-specific policies to school type. For each regression, all variables of interest were entered in a single step. In a final, hierarchical regression, only significant policies and practices identified in the first two regression models (Step 2) were included alongside personal and school characteristics, which served as controls (Step 1).

## 4. Results

### 4.1. Relationship Between Policies and Practices and Social Worker-Reported School Type

Chi-square tests of independence found several significant relationships between type of school (i.e., schools identified as trauma informed or not) and policies present (see Table 3). In fact, schools identified as trauma informed were statistically more likely to have 24 of the 33 policies or practices surveyed. For example, trauma training was present in 80.5% of trauma-informed schools and in 33.1% of non-trauma-informed schools, χ^2^(1, 530) = 98.95, *p* < 0.001. Resources for STS and self-care, trauma psychoeducation for students or parents, screening for trauma symptoms, and trauma interventions or treatments (e.g., CBITS) were all more prevalent in trauma-informed schools. Unexpectedly, metal detectors were also more common in trauma-informed schools: 16.9% of trauma-informed schools had them vs. 5.3% of non-trauma-informed schools, χ^2^(1, 530) = 18.62, *p* < 0.001. Several COVID-19-related policies were more common in trauma-informed schools, including providing for students’ and families’ basic needs, χ^2^(1, 530) = 17.30, *p* < 0.001, and guidelines for appropriate parent communication/behaviors, χ^2^(1, 530) = 14.45, *p* < 0.001.

### 4.2. Policies Associated with the Identification of a School as Trauma Informed

Multiple logistic regressions were performed to determine whether the presence of certain policies and practices in a school could point to whether respondents would identify it as trauma informed. Regression analyses began with comparisons of policies and practices to school type (trauma-informed or non-trauma-informed). A cutoff value of 0.3 was used to reflect the likelihood of a positive outcome in the sample.

The first model, which compared each of 18 general school policies with school type, was significant, χ^2^(18) = 159.17, *p* < 0.001, with a Nagelkerke *R*-squared of 0.37 (see Table 4). Trauma training was 6.3 times more likely in schools identified as trauma informed (*p* < 0.001); resources for STS and self-care were 1.8 times more likely in a trauma-informed school (*p* < 0.05); and metal detectors were 4.1 times more likely in a trauma-informed school (*p* < 0.001).

A second regression comparing 14 COVID-19-related policies with school type was also significant, χ^2^(14) = 44.36, *p* < 0.001, with a Nagelkerke *R*-squared of 0.11. In the second model (see Table 5), providing for students’ and families’ basic needs was associated with whether a school was identified as trauma informed. Providing for students’ and families’ basic needs was 2.1 times more likely in a trauma-informed school (*p* < 0.01).

The third model, a hierarchical logistic regression (see Table 6), entered all respondent demographics (i.e., years of experience, gender, and race and ethnicity) and school characteristics (i.e., urbanicity, grade level, region, and percentage of students who qualify for free or reduced-price lunch, are from marginalized populations, drop out, and enter college) in Step 1 and significant policies identified in prior regressions in Step 2. The final model was also significant, χ^2^(26) = 138.70, *p* < 0.001. This model correctly classified 85.3% of trauma-informed schools and 76.4% of non-trauma-informed schools, for an average of 79.2% accuracy. A nonsignificant Hosmer–Lemeshow goodness-of-fit test and Nagelkerke *R*-squared of 0.51 indicated that the model was a good fit for the data and accounted for a reasonable percentage of variance [74]. Significant policies associated with schools identified as trauma informed were the presence of trauma training for staff (*OR* = 21.17, 95% CI [8.60, 52.15], *p* < 0.001) and resources for STS and self-care (*OR* = 5.36, 95% CI [2.258, 12.710], *p* < 0.001).

As might be expected, trauma-informed schools were more likely to have trauma training and STS resources. Establishing the connection between training and the identification of a school as trauma informed is important because it is possible that schools perceive trauma-informed interventions in an overarching school safety approach. As demonstrated in the first and second regressions, schools reported an array of interventions and trainings in trauma-informed schools that are both trauma aware and law enforcement-related. However, our findings from the third regression show that after controlling for personal and school characteristics, trauma-informed schools were significantly more likely to have only trauma training and STS resources.

No school characteristics were found to be significantly associated with whether a school was identified as trauma informed. However, the number of years a respondent had worked as a school social worker was associated with whether they reported working in a trauma-informed school. All social workers who had more than 1 year of experience were less likely to report working in a trauma-informed school. This could mean that new social workers are more likely to start their careers in higher-need schools with more trauma-informed services. Given the high turnover rate in lower-resourced schools, it is possible that over time, school social workers migrate to schools with more resources.

## 5. Discussion

This was the first study to evaluate policy and practice differences between U.S. schools identified as trauma informed and those that were not. The study also highlights the views of school social workers—school personnel commonly tasked with responding to trauma and other mental health needs of students, families, and staff [20,21]. Social worker views have often been excluded from the evaluation of trauma-informed approaches in schools; thus, this article presents a critical and often overlooked perspective.

There were clear differences in policies and practices between schools identified as trauma informed and not. Many policy differences may be expected (e.g., increased prevalence of trauma training and resources for STS in trauma-informed schools). However, other findings were more surprising: Although uncommon in schools overall, metal detectors were present in schools identified as trauma informed at more than three times the rate in non-trauma-informed schools. In fact, every policy and practice we surveyed was more common in schools identified as trauma informed. This means that trauma-informed schools tend to enact law enforcement strategies alongside trauma-informed and other supportive practices. It is possible that some differences between trauma-informed and non-trauma-informed schools relate more to the availability and allocation of resources rather than to strategic, mission-driven policy and practice decisions. Future research should explore this possibility.

We also suggest additional consideration of the conceptualization of trauma-informed approaches in schools and their required components. In recent years, many evidence-based programs have become aligned with a trauma-informed approach [16,37,38,39]. However, it is unclear to what extent these programs’ strategies relate to the intentions and conceptualizations of trauma-informed interventions.

In our initial regression models, four school policies were significantly associated with whether a school was identified as trauma informed: (1) trauma training, (2) metal detectors, (3) providing for students’ and families’ basic needs, and (4) resources for STS. However, when personal demographics and school characteristics were held constant, only the presence of trauma training and resources for STS were associated with schools identified as trauma informed. The relationships between metal detectors, providing for basic needs, and school type (trauma informed or not) were accounted for by other factors.

Both identification as a trauma-informed school and the presence of metal detectors were related to the proportion of students who qualified for free or reduced-price lunch and who were from historically marginalized populations. Prior research [65,75] noted similar connections. Additional research is thus suggested to assess whether differences in characteristics between schools identified as trauma informed and not are circumstantial or intentional.

Prior research has shown that schools tend to limit their trauma-informed approaches to providing trauma training and access to trauma-specific clinical treatments for students [19]. Our findings suggest this may still be true. If so, we must consider whether training and clinical treatments are sufficient to create a trauma-informed school. Alternatively, incorporating just these two programmatic elements may limit schools’ ability to achieve the goals of a trauma-informed approach: creating a safer, more supportive school environment where children of all backgrounds can succeed. Future research should explore this possibility.

One personal characteristic of respondents was also significant in the final model: years of service as a school social worker. We found that school social workers with more experience were less likely to work in schools they identified as trauma informed. This finding could be the result of several factors. Less experienced social workers may seek out more supportive work environments, as would be expected of trauma-informed schools. Another explanation that future research should explore is that there may be higher turnover of school social workers in high-trauma settings. It is also possible that social workers with more experience chose to migrate to schools with less trauma, perhaps to avoid burnout or for other reasons.

In sum, our findings are valuable because to date, it has been unclear whether any differences exist between schools identified as trauma informed and those that were not, despite implementation suggestions for trauma-informed approaches from SAMHSA [76] and others. Trauma-informed approaches in schools would be expected to include many more components, including organizational policy changes and the availability of trauma-specific screening and treatments [16,26,33,34]. The continued conceptualization of trauma-informed schools should also integrate ideas from the organizational literature.

Going forward, it will be critical to answer larger questions associated with implementing trauma-informed approaches in schools. We must assess whether such approaches support or detract from children’s educational experiences and outcomes. This will require first building consensus about the expected outcomes. It is also important to consider whether trauma-informed approaches could be seamlessly integrated in the broader mission of schools without identifying schools as trauma informed per se. Perhaps the key components of a trauma-informed approach can be integrated into existing school models such as community schools or welcoming, empowering, and monitoring approaches [8,11]. These considerations are part of a broader debate on the purpose of schools, which was heightened during the COVID-19 pandemic [77,78]. Without a public and academic discussion on the purpose of schools, many will disagree that trauma-informed approaches should even be in schools [19,79]. In the United States, such issues have been a major focus of recent educational policy disagreements at the school board and state levels [80]. As a result, we recommend greater conceptual, policy, and public debate so that trauma-informed approaches will not appear to be competing with the academic purpose of schools.

## 6. Strengths, Limitations, and Future Research

This study was the first to compare actual policy and practice differences between U.S. schools identified as trauma informed and not trauma informed. The findings represent a national convenience sample. Survey respondents practiced in all but seven states and their demographics generally matched what we know about the U.S. population of school social workers [81,82]. However, some states were represented by a handful of respondents. Further, the demographics of school social workers nationally prevented the analysis of some racial, ethnic, and gender identity groups because of their low numbers in the profession overall. Future studies should oversample and recruit respondents from these groups.

A limitation of all research of trauma-informed schools currently is the lack of formal measurements, conceptual designations, or a process to identify schools that are trauma informed. Given the nascency of this field, we expect many future developments in these areas. Additional research is needed to understand the phenomenon of trauma-informed schools and their impact on school staff, students, and families. Researchers should seek to understand the intentionality of trauma-informed approaches and evaluate whether the approaches as implemented are benefiting or detracting from children’s school experiences and outcomes. Future research should also seek to understand to what extent policy or practice differences are intentional and whether they are believed to be an essential component of a trauma-informed approach. Qualitative interviews and observations would be supportive of this goal. Case studies that describe ideal trauma-informed schools are also needed. We suggest an analysis of the resource allocations between trauma-informed and non-trauma-informed schools to understand why trauma-informed schools were more likely to have every policy we surveyed as well as higher percentages of students from minoritized populations and who qualify for free or reduced-price lunch.

To date, much of the research on trauma-informed schools has focused on the outcomes of a specific intervention [26,27]. However, nothing in the conceptual models for trauma-informed approaches indicates that a program or intervention is essential to create such an environment [16,33]. Furthermore, the emphasis on school programming instead of a broader organizational focus creates fragmented solutions that cannot address the needs of the whole school or community [6,8].

## Figures and Tables

**Table 1:** Participant characteristics (*N* = 538).

Characteristic	*n*	%
Gender		
Male	39	7.2
Female	488	90.7
Gender nonconforming	2	0.4
Other or prefer not to answer	9	1.7
Race and ethnicity		
Asian American	4	0.7
Black or African American	40	7.4
Native Hawaiian or Pacific Islander	1	0.2
Hispanic or Latinx	63	11.7
Native American or Alaska Native	1	0.2
White or Caucasian	391	72.7
Multiracial	23	4.3
Other or no answer	15	2.8
Role		
School social worker	480	89.2
District supervisor	8	1.5
Head of services	5	0.9
School-based contractor	3	0.6
Other or no answer	42	7.8
Years of experience		
<1	23	4.3
1–2	45	8.4
3–5	96	17.9
6–10	95	17.7
11–15	69	12.9
16–20	81	15.1
>20	127	23.7
Schools served ^a^		
Preschool	92	17.1
Elementary	310	57.6
Middle or junior high	260	48.3
High	241	44.8
Alternative	69	12.8
Other	7	1.3

^a^ Participants could select more than one.

**Table 2:** School characteristics reported by participants.

Characteristic	*n*	%	*M*	*SD*
Settings				
Urban	205	38.1		
Suburban	231	43.3		
Rural	97	18.2		
U.S. region				
Northeast	110	20.5		
Midwest	191	35.6		
South	101	18.9		
West	134	25.0		
Students (%)				
Receiving free or reduced-price lunch			63.8	28.4
Historically marginalized populations			55.7	30.7
Drop out			19.5	18.1
Enter college			55.5	22.1
Grade level				
Preschool	6	1.5		
Elementary	156	38.3		
Middle or junior high	106	26.0		
High	139	34.2		

*Note*. Means and standard deviations in the table refer to the percentage of students reported by school social workers to reflect each characteristic.

**Table 3:** Policies and practices in schools identified by social workers as trauma informed (TI) and not (NTI).

Policy or Practice	TI (*n* = 154) *n* (%)	NTI (*n* = 378) *n* (%)	χ^2^_(df = 1)_	φ
General (α = 0.699)				
Trauma training for staff	124 (80.5)	125 (33.1)	98.95 ***	0.431
Resources for STS and self-care	54 (35.1)	43 (11.4)	41.19 ***	0.278
Trauma psychoeducation for students or parents	38 (24.7)	24 (6.3)	35.79 ***	0.259
Classroom practices that help de-escalate and refocus students	64 (61.0)	132 (34.9)	30.55 ***	0.240
Screening for trauma symptoms or PTSD	28 (18.2)	18 (4.8)	24.95 ***	0.217
Trauma interventions or treatments (e.g., CBITS)	47 (30.5)	49 (13.0)	22.81 ***	0.207
Metal detectors	26 (16.9)	20 (5.3)	18.62 ***	0.187
Teaching materials reflect diverse students	87 (56.5)	137 (36.2)	18.41 ***	0.186
Screening for adverse childhood experiences	23 (14.9)	21 (5.6)	12.69 ***	0.154
Restorative justice practices	89 (57.8)	157 (41.5)	11.63 ***	0.148
Equitable school discipline	66 (42.9)	105 (27.8)	11.41 ***	0.146
Commitment to creating a safe, supportive learning environment for all students	136 (88.3)	285 (75.4)	11.05 ***	0.144
School climate programs	75 (48.7)	127 (33.6)	10.60 **	0.141
Modifying the curriculum to remove triggering content	21 (13.6)	25 (6.6)	6.83 **	0.113
SEL skills training	131 (85.1)	288 (76.2)	5.15 *	0.098
MTSS (including PBIS)	127 (82.5)	287 (75.9)	2.71	0.071
Student searches	29 (18.8)	51 (13.5)	2.44	0.068
None of the above	0 (0.0)	4 (1.1)	1.64	0.056
School resource officers	83 (53.9)	197 (52.1)	0.14	0.016
COVID-19-related (α = 0.729)				
Providing for students’ and families’ basic needs	123 (79.9)	231 (61.1)	17.30 ***	0.180
Guidelines for appropriate parent communication and behaviors	50 (32.5)	66 (17.5)	14.45 ***	0.165
Support for struggling students	103 (66.9)	198 (52.4)	9.37 **	0.133
Hiring new teachers or support staff	50 (32.5)	77 (20.4)	8.81 **	0.129
Online or hybrid school options	70 (45.5)	125 (33.1)	7.23 **	0.117
Regular COVID-19 testing	73 (47.4)	132 (34.9)	7.20 **	0.116
Opening or closing schools based on COVID-19 case rates	58 (37.7)	101 (26.7)	6.25 *	0.108
Mandatory vaccination	27 (17.5)	37 (9.8)	6.20 *	0.108
Counseling for teachers	23 (14.9)	31 (8.2)	5.44 *	0.101
Consistent use of masks	79 (51.3)	168 (44.4)	2.07	0.062
Social distancing	63 (40.9)	136 (36.0)	1.14	0.046
Expanded teaching hours	11 (7.1)	19 (5.0)	0.92	0.042
Hiring new mental health professionals	46 (29.9)	106 (28.0)	0.18	0.018
Requiring students to quarantine after possible exposure	103 (66.9)	246 (65.1)	0.16	0.017

* *p* < 0.05. ** *p* < 0.01. *** *p* < 0.001.

**Table 4:** General school policies and practices associated with social worker identification of a school as trauma informed.

Policy or Characteristic				95% CI				
	*B*	*SE*	*Exp*(*B*)	*LL*	*UL*	*Wald*	*df*	*p*
Trauma training for staff *	1.84	0.26	6.30	3.81	10.43	51.39	1	<0.001
Metal detectors *	1.41	0.41	4.10	1.83	9.18	11.75	1	<0.001
Resources for STS and self-care *	0.61	0.29	1.85	1.04	3.28	4.44	1	0.035
School climate programs	0.44	0.24	1.55	0.96	2.50	3.21	1	0.073
Classroom practices that help de-escalate and refocus students	0.45	0.25	1.57	0.95	2.57	3.13	1	0.077
Trauma psychoeducation for students or parents	0.56	0.34	1.75	0.90	3.40	2.71	1	0.100
Teaching materials that reflect diverse students	0.37	0.26	1.44	0.87	2.39	2.02	1	0.156
Restorative justice practices	0.32	0.24	1.37	0.86	2.18	1.76	1	0.184
Screening for trauma symptoms or PTSD	0.40	0.43	1.49	0.64	3.48	0.87	1	0.352
Screening for adverse childhood experiences	0.40	0.43	1.49	0.64	3.43	0.86	1	0.354
Equitable school discipline	−0.19	0.27	0.83	0.50	1.340	0.49	1	0.486
Commitment to creating a safe, supportive learning environment for all students	0.24	0.34	1.26	0.65	2.46	0.48	1	0.490
Student searches	−0.23	0.34	0.79	0.41	1.55	0.46	1	0.499
MTSS, including PBIS	−0.19	0.30	0.83	0.46	1.50	0.39	1	0.534
School resource officers	0.13	0.24	1.14	0.71	1.81	0.29	1	0.589
SEL skills training	−0.14	0.33	0.87	0.46	1.64	0.20	1	0.657
Modifying the curriculum to remove triggering content	0.14	0.39	1.15	0.54	2.47	0.13	1	0.721
Trauma interventions or treatments (e.g., CBITS, SSET, TF-CBT)	−0.06	0.30	0.95	0.52	1.72	0.03	1	0.857
Constant *	−3.02	0.43	0.05			48.87	1	<0.001
Model χ^2^	159.17							
Nagelkerke *R*^2^	0.37							
*n*	532							

*Note.* Outcome variable was whether a school was identified by school social workers as being trauma informed. * Significant predictor.

**Table 5:** COVID-19-related policies and practices associated with social worker identification of a school as trauma informed.

Policy or Characteristic				95% CI				
	*B*	*SE*	*Exp*(*B*)	*LL*	*UL*	*Wald*	*df*	*p*
Providing for students’ and families’ basic needs (e.g., food, technology) *	0.75	0.25	2.12	1.29	3.47	8.83	1	0.003
Guidelines for appropriate parent communication or behaviors	0.46	0.25	1.59	0.97	2.60	3.34	1	0.068
Hiring new mental health professionals	−0.40	0.25	0.673	0.41	1.10	2.55	1	0.111
Requiring students to quarantine after possible exposure	−0.39	0.25	0.68	0.42	1.10	2.45	1	0.118
Mandatory vaccination	0.47	0.30	1.60	0.88	2.91	2.41	1	0.120
Regular COVID-19 testing	0.35	0.23	1.42	0.91	2.23	2.34	1	0.126
Hiring new teachers or support staff	0.36	0.25	1.43	0.88	2.34	2.09	1	0.148
Opening or closing schools based on COVID-19 case rates	0.28	0.23	1.33	0.85	2.07	1.57	1	0.211
Online or hybrid school options	0.26	0.22	1.30	0.84	2.01	1.36	1	0.243
Support for struggling students	0.19	0.23	1.21	0.77	1.91	0.71	1	0.400
Social distancing	−0.15	0.27	0.86	0.51	1.46	0.30	1	0.583
Counseling for teachers	0.16	0.34	1.17	0.60	2.28	0.22	1	0.643
Expanded teaching hours	0.08	0.43	1.08	0.47	2.48	0.03	1	0.860
Consistent use of masks	0.03	0.27	1.03	0.61	1.74	0.02	1	0.904
Constant *	−1.77	0.25	0.17			48.65	1	<0.001
Model χ^2^	44.36							
Pseudo *R*^2^	0.11							
*n*	532							

*Note.* Outcome variable was whether a school was identified by school social workers as being trauma informed. * Significant predictor.

**Table 6:** Hierarchical logistic regression for identifying schools social workers claim are trauma informed by personal demographics, school characteristics, and policies and practices.

Policy or Characteristic	Step 1				Step 2			
	*B*	*SE*	*OR*	95% CI	*B*	*SE*	*OR*	95% CI
Constant	1.018	1.663	2.767		−2.276	1.996	0.103	
Gender (ref = male)	−0.736	0.454	0.479	0.197, 1.167	−0.772	0.553	0.462	0.156, 1.366
Race and ethnicity (ref = other)								
Black or African American	0.225	0.820	1.253	0.251, 6.255	0.449	0.963	1.567	0.237, 10.346
Hispanic	0.348	0.680	1.417	0.374, 5.370	0.126	0.813	1.134	0.230, 5.587
White	0.369	0.596	1.447	0.450, 4.652	0.194	0.689	1.215	0.314, 4.692
Participant years of experience (<1 = Ref)								
1–2	−1.150	0.770	0.317	0.070, 1.432	−2.105	0.891	0.122	0.021, 0.699
3–5	−0.368	0.673	0.692	0.185, 2.589	−2.321	0.807	0.098 *	0.020, 0.478
6–10	−1.093	0.692	0.335	0.086, 1.300	−2.961	0.844	0.052 **	0.010, 0.271
11–15	−1.452	0.736	0.234	0.055, 0.991	−2.897	0.891	0.055 *	0.010, 0.316
16–20	−1.443	0.721	0.236	0.057, 0.971	−3.972	0.896	0.019 **	0.003, 0.109
20+	−1.452	0.683	0.234	0.061, 0.892	−3.316	0.825	0.036 **	0.007, 0.183
Students								
Qualify for free or reduced-price lunch	−0.007	0.008	0.993	0.978, 1.009	0.003	0.009	1.003	0.985, 1.021
From historically marginalized populations	0.011	0.007	1.011	0.996, 1.026	0.021	0.010	1.021	1.001, 1.041
Drop out	0.000	0.010	1.000	0.0981, 1.019	0.005	0.013	1.005	0.979, 1.032
Enter college	−0.007	0.008	0.993	0.979, 1.008	−0.001	0.010	0.999	0.980, 1.019
School setting (ref = urban)								
Suburban	−0.353	0.372	0.703	0.339, 1.456	0.375	0.489	1.456	0.558, 3.795
Rural	0.074	0.415	1.077	0.478, 2.428	0.556	0.542	1.744	0.603, 5.049
Grade level (ref = preschool)								
Elementary	−0.479	1.009	0.619	0.086, 4.473	0.249	1.103	1.282	0.148, 11.137
Middle or junior	0.114	1.032	1.120	0.148, 8.469	0.684	1.137	1.982	0.223, 18.403
High school	−0.129	1.012	0.879	0.121, 6.393	0.373	1.115	1.452	0.163, 12.909
U.S. region (ref = Northeast)								
Midwest	0.605	0.375	1.831	0.878, 3.817	0.648	0.485	1.913	0.739, 4.952
South	−0.096	0.444	0.908	0.380, 2.168	−0.372	0.555	0.689	0.232, 2.047
West	−0.100	0.439	0.905	0.383, 2.141	−0.277	0.577	0.758	0.245, 2.384
Trauma training for staff					3.053	0.460	21.172 **	8.595, 52.151
Resources for STS and self-care					1.678	0.441	5.357 **	2.258, 12.710
Metal detectors					1.105	0.610	3.018	0.914, 9.967
Providing for students’ and families’ basic needs					0.408	0.393	1.503	0.696, 3.245
Model χ^2^ (*df*)	29.752 (22)				138.696 (26) **			
Δχ^2^ (*df*)					108.944 (3) **			
Nagelkerke *R*^2^	0.13				0.51			
*n*	307							

*Note.* Other races and ethnicities include Asian, Native Hawaiian, Pacific Islander, Native American, and multiracial. The outcome variable was whether school social workers identified a school as being trauma informed. * *p* < 0.01. ** *p* < 0.001.

## Data Availability

Data are contained within the article.
